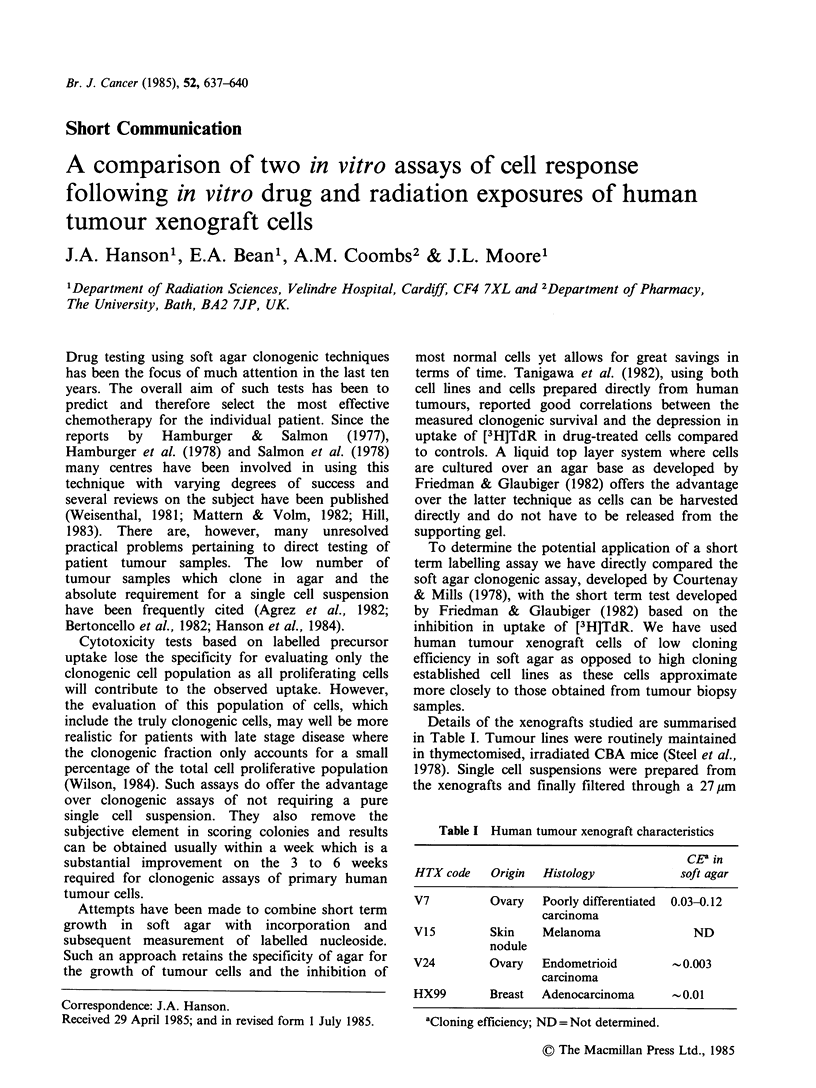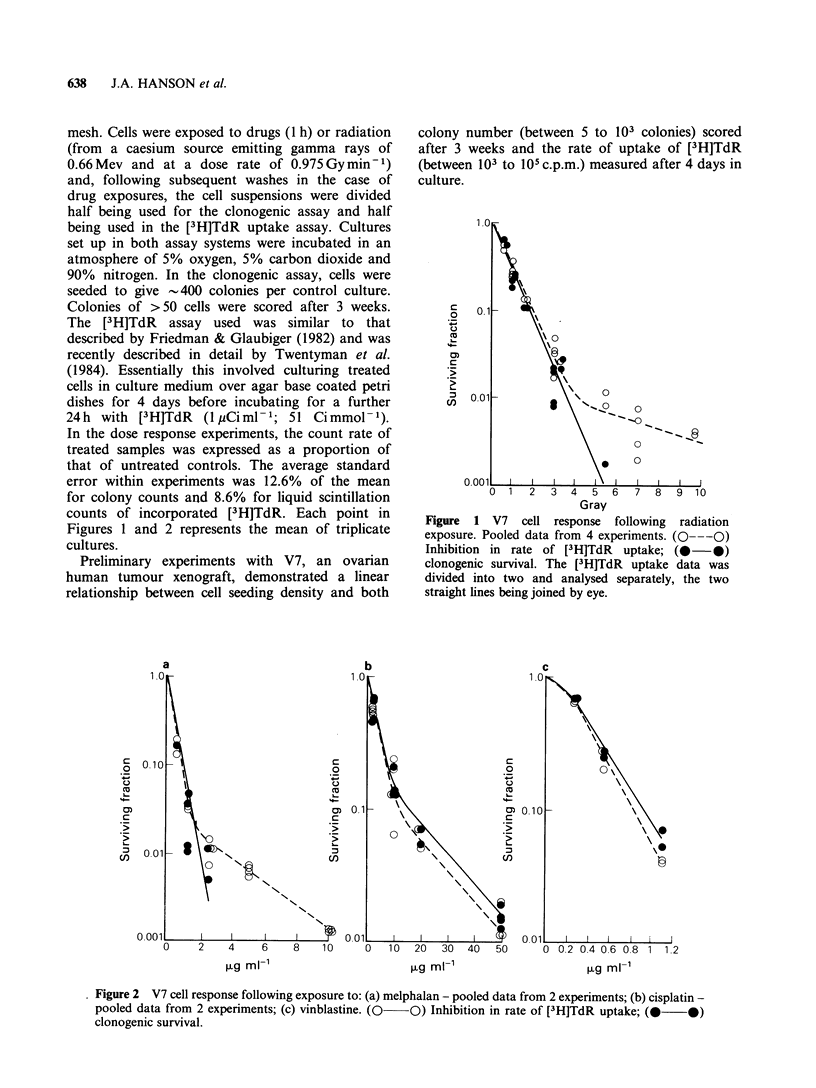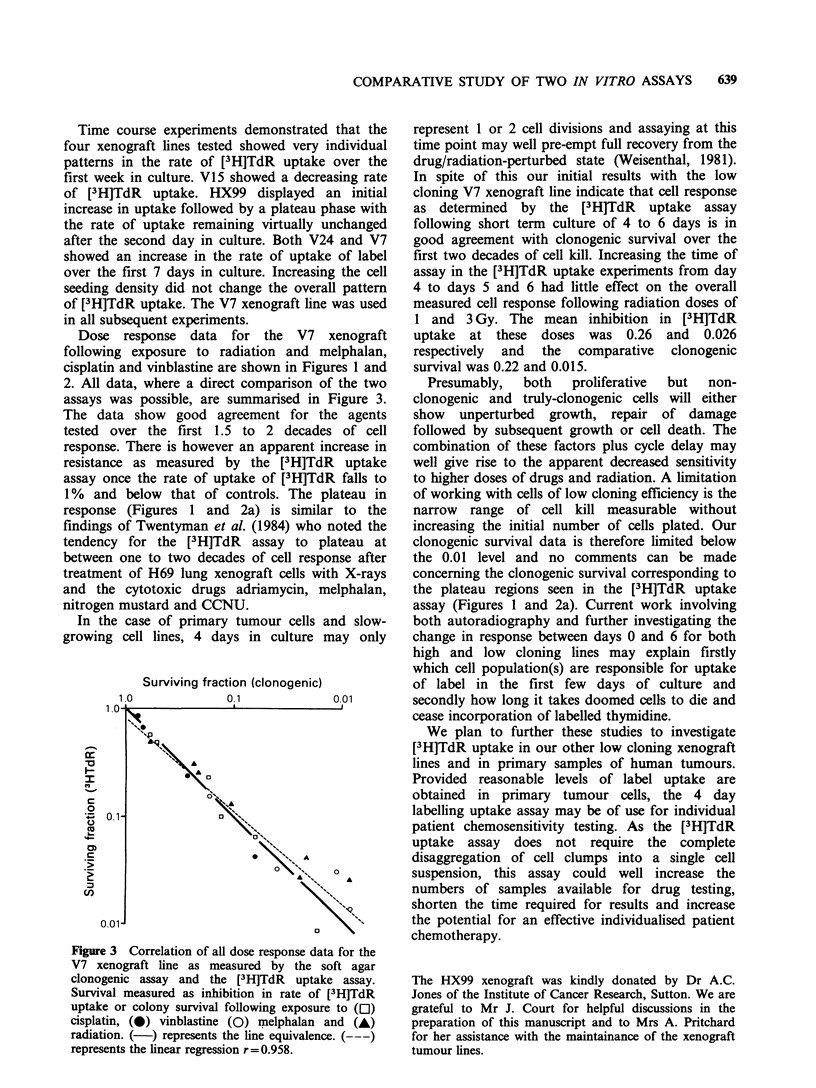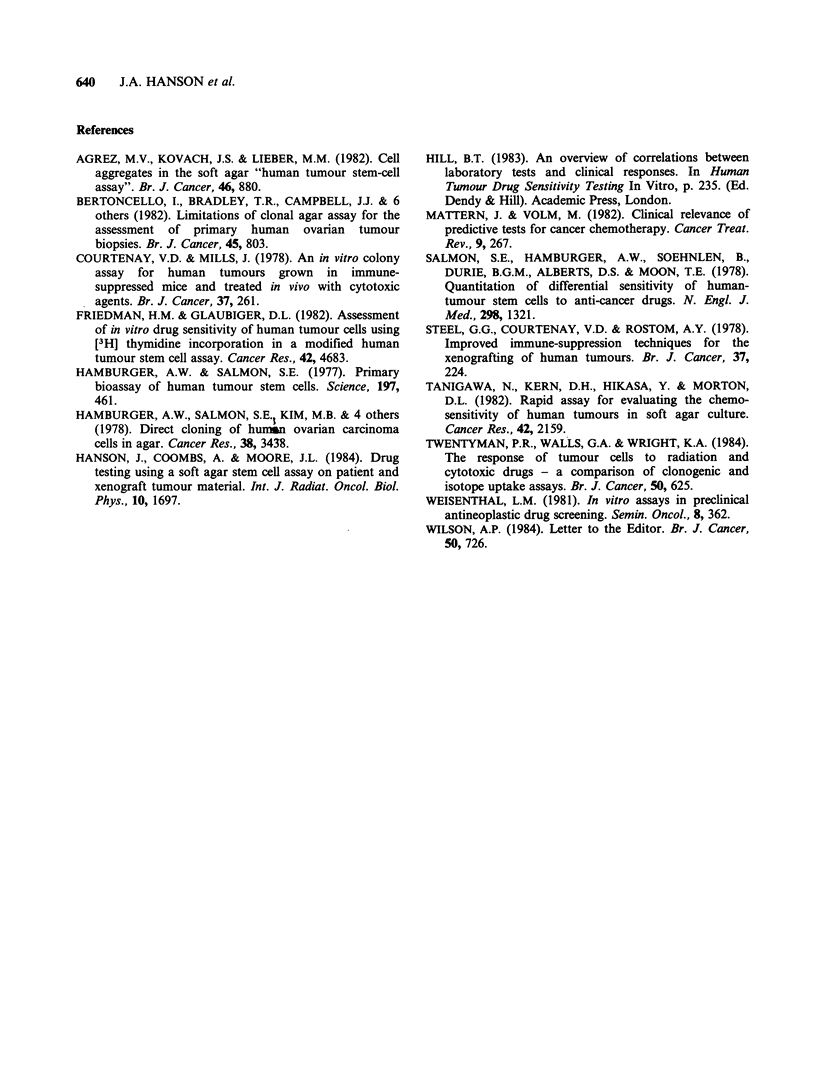# A comparison of two in vitro assays of cell response following in vitro drug and radiation exposures of human tumour xenograft cells.

**DOI:** 10.1038/bjc.1985.239

**Published:** 1985-10

**Authors:** J. A. Hanson, E. A. Bean, A. M. Coombs, J. L. Moore


Br. J. Cancer (1985), 52, 637–640

### Short Communication

### A comparison of two *in vitro* assays of cell response following *in vitro* drug and radiation exposures of human tumour xenograft cells

J.A. Hanson[1], E.A. Bean[1], A.M. Coombs[2] & J.L. Moore[1]

[1]*Department of Radiation Sciences, Velindre Hospital, Cardiff, CF4 7XL and* [2]*Department of Pharmacy, The University, Bath, BA2 7JP, UK.*

Drug testing using soft agar clonogenic techniques has been the focus of much attention in the last ten years. The overall aim of such tests has been to predict and therefore select the most effective chemotherapy for the individual patient. Since the reports by Hamburger & Salmon (1977), Hamburger *et al.* (1978) and Salmon *et al.* (1978) many centres have been involved in using this technique with varying degrees of success and several reviews on the subject have been published (Weisenthal, 1981; Mattern & Volm, 1982; Hill, 1983). There are, however, many unresolved practical problems pertaining to direct testing of patient tumour samples. The low number of tumour samples which clone in agar and the absolute requirement for a single cell suspension have been frequently cited (Agrez *et al.*, 1982; Bertoncello *et al.*, 1982; Hanson *et al.*, 1984).

Cytotoxicity tests based on labelled precursor uptake lose the specificity for evaluating only the clonogenic cell population as all proliferating cells will contribute to the observed uptake. However, the evaluation of this population of cells, which include the truly clonogenic cells, may well be more realistic for patients with late stage disease where the clonogenic fraction only accounts for a small percentage of the total cell proliferative population (Wilson, 1984). Such assays do offer the advantage over clonogenic assays of not requiring a pure single cell suspension. They also remove the subjective element in scoring colonies and results can be obtained usually within a week which is a substantial improvement on the 3 to 6 weeks required for clonogenic assays of primary human tumour cells.

Attempts have been made to combine short term growth in soft agar with incorporation and subsequent measurement of labelled nucleoside. Such an approach retains the specificity of agar for the growth of tumour cells and the inhibition of most normal cells yet allows for great savings in terms of time. Tanigawa *et al.* (1982), using both cell lines and cells prepared directly from human tumours, reported good correlations between the measured clonogenic survival and the depression in uptake of [$^3$H]TdR in drug-treated cells compared to controls. A liquid top layer system where cells are cultured over an agar base as developed by Friedman & Glaubiger (1982) offers the advantage over the latter technique as cells can be harvested directly and do not have to be released from the supporting gel.

To determine the potential application of a short term labelling assay we have directly compared the soft agar clonogenic assay, developed by Courtenay & Mills (1978), with the short term test developed by Friedman & Glaubiger (1982) based on the inhibition in uptake of [$^3$H]TdR. We have used human tumour xenograft cells of low cloning efficiency in soft agar as opposed to high cloning established cell lines as these cells approximate more closely to those obtained from tumour biopsy samples.

Details of the xenografts studied are summarised in Table I. Tumour lines were routinely maintained in thymectomised, irradiated CBA mice (Steel *et al.*, 1978). Single cell suspensions were prepared from the xenografts and finally filtered through a 27 $\mu$m

**Table I** Human tumour xenograft characteristics

| *HTX code* | *Origin* | *Histology* | *CE*[a] *in soft agar* |
|---|---|---|---|
| V7 | Ovary | Poorly differentiated carcinoma | 0.03–0.12 |
| V15 | Skin nodule | Melanoma | ND |
| V24 | Ovary | Endometrioid carcinoma | ~0.003 |
| HX99 | Breast | Adenocarcinoma | ~0.01 |

[a]Cloning efficiency; ND = Not determined.

Correspondence: J.A. Hanson.
Received 29 April 1985; and in revised form 1 July 1985.

© The Macmillan Press Ltd., 1985

mesh. Cells were exposed to drugs (1 h) or radiation (from a caesium source emitting gamma rays of 0.66 Mev and at a dose rate of 0.975 Gy min$^{-1}$) and, following subsequent washes in the case of drug exposures, the cell suspensions were divided half being used for the clonogenic assay and half being used in the [$^3$H]TdR uptake assay. Cultures set up in both assay systems were incubated in an atmosphere of 5% oxygen, 5% carbon dioxide and 90% nitrogen. In the clonogenic assay, cells were seeded to give ~400 colonies per control culture. Colonies of >50 cells were scored after 3 weeks. The [$^3$H]TdR assay used was similar to that described by Friedman & Glaubiger (1982) and was recently described in detail by Twentyman *et al.* (1984). Essentially this involved culturing treated cells in culture medium over agar base coated petri dishes for 4 days before incubating for a further 24 h with [$^3$H]TdR (1 $\mu$Ci ml$^{-1}$; 51 Ci mmol$^{-1}$). In the dose response experiments, the count rate of treated samples was expressed as a proportion of that of untreated controls. The average standard error within experiments was 12.6% of the mean for colony counts and 8.6% for liquid scintillation counts of incorporated [$^3$H]TdR. Each point in Figures 1 and 2 represents the mean of triplicate cultures.

Preliminary experiments with V7, an ovarian human tumour xenograft, demonstrated a linear relationship between cell seeding density and both colony number (between 5 to $10^3$ colonies) scored after 3 weeks and the rate of uptake of [$^3$H]TdR (between $10^3$ to $10^5$ c.p.m.) measured after 4 days in culture.

**Figure 1** V7 cell response following radiation exposure. Pooled data from 4 experiments. (○---○) Inhibition in rate of [$^3$H]TdR uptake; (●——●) clonogenic survival. The [$^3$H]TdR uptake data was divided into two and analysed separately, the two straight lines being joined by eye.

**Figure 2** V7 cell response following exposure to: (a) melphalan – pooled data from 2 experiments; (b) cisplatin – pooled data from 2 experiments; (c) vinblastine. (○——○) Inhibition in rate of [$^3$H]TdR uptake; (●——●) clonogenic survival.

Time course experiments demonstrated that the four xenograft lines tested showed very individual patterns in the rate of [$^3$H]TdR uptake over the first week in culture. V15 showed a decreasing rate of [$^3$H]TdR uptake. HX99 displayed an initial increase in uptake followed by a plateau phase with the rate of uptake remaining virtually unchanged after the second day in culture. Both V24 and V7 showed an increase in the rate of uptake of label over the first 7 days in culture. Increasing the cell seeding density did not change the overall pattern of [$^3$H]TdR uptake. The V7 xenograft line was used in all subsequent experiments.

Dose response data for the V7 xenograft following exposure to radiation and melphalan, cisplatin and vinblastine are shown in Figures 1 and 2. All data, where a direct comparison of the two assays was possible, are summarised in Figure 3. The data show good agreement for the agents tested over the first 1.5 to 2 decades of cell response. There is however an apparent increase in resistance as measured by the [$^3$H]TdR uptake assay once the rate of uptake of [$^3$H]TdR falls to 1% and below that of controls. The plateau in response (Figures 1 and 2a) is similar to the findings of Twentyman *et al.* (1984) who noted the tendency for the [$^3$H]TdR assay to plateau at between one to two decades of cell response after treatment of H69 lung xenograft cells with X-rays and the cytotoxic drugs adriamycin, melphalan, nitrogen mustard and CCNU.

In the case of primary tumour cells and slow-growing cell lines, 4 days in culture may only represent 1 or 2 cell divisions and assaying at this time point may well pre-empt full recovery from the drug/radiation-perturbed state (Weisenthal, 1981). In spite of this our initial results with the low cloning V7 xenograft line indicate that cell response as determined by the [$^3$H]TdR uptake assay following short term culture of 4 to 6 days is in good agreement with clonogenic survival over the first two decades of cell kill. Increasing the time of assay in the [$^3$H]TdR uptake experiments from day 4 to days 5 and 6 had little effect on the overall measured cell response following radiation doses of 1 and 3 Gy. The mean inhibition in [$^3$H]TdR uptake at these doses was 0.26 and 0.026 respectively and the comparative clonogenic survival was 0.22 and 0.015.

Presumably, both proliferative but non-clonogenic and truly-clonogenic cells will either show unperturbed growth, repair of damage followed by subsequent growth or cell death. The combination of these factors plus cycle delay may well give rise to the apparent decreased sensitivity to higher doses of drugs and radiation. A limitation of working with cells of low cloning efficiency is the narrow range of cell kill measurable without increasing the initial number of cells plated. Our clonogenic survival data is therefore limited below the 0.01 level and no comments can be made concerning the clonogenic survival corresponding to the plateau regions seen in the [$^3$H]TdR uptake assay (Figures 1 and 2a). Current work involving both autoradiography and further investigating the change in response between days 0 and 6 for both high and low cloning lines may explain firstly which cell population(s) are responsible for uptake of label in the first few days of culture and secondly how long it takes doomed cells to die and cease incorporation of labelled thymidine.

We plan to further these studies to investigate [$^3$H]TdR uptake in our other low cloning xenograft lines and in primary samples of human tumours. Provided reasonable levels of label uptake are obtained in primary tumour cells, the 4 day labelling uptake assay may be of use for individual patient chemosensitivity testing. As the [$^3$H]TdR uptake assay does not require the complete disaggregation of cell clumps into a single cell suspension, this assay could well increase the numbers of samples available for drug testing, shorten the time required for results and increase the potential for an effective individualised patient chemotherapy.

**Figure 3** Correlation of all dose response data for the V7 xenograft line as measured by the soft agar clonogenic assay and the [$^3$H]TdR uptake assay. Survival measured as inhibition in rate of [$^3$H]TdR uptake or colony survival following exposure to (□) cisplatin, (●) vinblastine (○) melphalan and (▲) radiation. (——) represents the line equivalence. (– – –) represents the linear regression $r = 0.958$.

The HX99 xenograft was kindly donated by Dr A.C. Jones of the Institute of Cancer Research, Sutton. We are grateful to Mr J. Court for helpful discussions in the preparation of this manuscript and to Mrs A. Pritchard for her assistance with the maintainance of the xenograft tumour lines.

### References

AGREZ, M.V., KOVACH, J.S. & LIEBER, M.M. (1982). Cell aggregates in the soft agar "human tumour stem-cell assay". *Br. J. Cancer*, **46**, 880.

BERTONCELLO, I., BRADLEY, T.R., CAMPBELL, J.J. & 6 others (1982). Limitations of clonal agar assay for the assessment of primary human ovarian tumour biopsies. *Br. J. Cancer*, **45**, 803.

COURTENAY, V.D. & MILLS, J. (1978). An *in vitro* colony assay for human tumours grown in immune-suppressed mice and treated *in vivo* with cytotoxic agents. *Br. J. Cancer*, **37**, 261.

FRIEDMAN, H.M. & GLAUBIGER, D.L. (1982). Assessment of *in vitro* drug sensitivity of human tumour cells using [$^3$H] thymidine incorporation in a modified human tumour stem cell assay. *Cancer Res.*, **42**, 4683.

HAMBURGER, A.W. & SALMON, S.E. (1977). Primary bioassay of human tumour stem cells. *Science*, **197**, 461.

HAMBURGER, A.W., SALMON, S.E., KIM, M.B. & 4 others (1978). Direct cloning of human ovarian carcinoma cells in agar. *Cancer Res.*, **38**, 3438.

HANSON, J., COOMBS, A. & MOORE, J.L. (1984). Drug testing using a soft agar stem cell assay on patient and xenograft tumour material. *Int. J. Radiat. Oncol. Biol. Phys.*, **10**, 1697.

HILL, B.T. (1983). An overview of correlations between laboratory tests and clinical responses. In *Human Tumour Drug Sensitivity Testing* In Vitro, p. 235. (Ed. Dendy & Hill). Academic Press, London.

MATTERN, J. & VOLM, M. (1982). Clinical relevance of predictive tests for cancer chemotherapy. *Cancer Treat. Rev.*, **9**, 267.

SALMON, S.E., HAMBURGER, A.W., SOEHNLEN, B., DURIE, B.G.M., ALBERTS, D.S. & MOON, T.E. (1978). Quantitation of differential sensitivity of human-tumour stem cells to anti-cancer drugs. *N. Engl. J. Med.*, **298**, 1321.

STEEL, G.G., COURTENAY, V.D. & ROSTOM, A.Y. (1978). Improved immune-suppression techniques for the xenografting of human tumours. *Br. J. Cancer*, **37**, 224.

TANIGAWA, N., KERN, D.H., HIKASA, Y. & MORTON, D.L. (1982). Rapid assay for evaluating the chemo-sensitivity of human tumours in soft agar culture. *Cancer Res.*, **42**, 2159.

TWENTYMAN, P.R., WALLS, G.A. & WRIGHT, K.A. (1984). The response of tumour cells to radiation and cytotoxic drugs – a comparison of clonogenic and isotope uptake assays. *Br. J. Cancer*, **50**, 625.

WEISENTHAL, L.M. (1981). *In vitro* assays in preclinical antineoplastic drug screening. *Semin. Oncol.*, **8**, 362.

WILSON, A.P. (1984). Letter to the Editor. *Br. J. Cancer*, **50**, 726.